# Effects of Family Doctor Contract Services on the Health-Related Quality of Life Among Individuals With Diabetes in China: Evidence From the CHARLS

**DOI:** 10.3389/fpubh.2022.865653

**Published:** 2022-05-04

**Authors:** Lingjie Wang, Wenbin Liu

**Affiliations:** School of Public Health, Fujian Medical University, Fuzhou, China

**Keywords:** family doctor contract services, diabetes, health-related quality of life, China Health and Retirement Longitudinal Study, difference-in-differences

## Abstract

**Background:**

Family doctor contract services (FDCS) has played a key role in diabetes management in China since 2016. The influence of FDCS on the physiological indexes of individuals with diabetes has been examined. However, little attention has been paid to its effect on the Health-Related Quality of Life (HRQoL). This study aims to fill this knowledge gap by evaluating the effect of FDCS on the HRQoL of individuals with diabetes.

**Methods:**

We identified 382 individuals with diabetes receiving all follow-up surveys in 2013, 2015, and 2018 from the China Health and Retirement Longitudinal Study (CHARLS). The HRQoL of the included individuals was estimated using results from the Short Form 36 (SF-36) questionnaire. The propensity score matching with the difference-in-differences (PSM-DID) approach was applied to quantify the effect of FDCS on the HRQoL among individuals with diabetes. A robust test was performed by setting the 2015 data as the treatment group for the placebo test.

**Results:**

The mean score of role-emotional (RE) increased from 54.25 to 61.63 among those who signed up to receive FDCS, while the corresponding score decreased from 57.77 to 51.04 among those who did not receive FDCS. Results from the regression analysis indicated that the use of FDCS was associated with significant improvement in RE (+14.10, *p* = 0.04) among individuals with diabetes. We did not find a statistically meaningful association between the FDCS and any of the other HRQoL domains: physical functioning (PF), role-physical (RP), bodily pain (BP), general health (GH), vitality (VT), social functioning (SF), and mental health (MH), respectively. The robustness analysis of the model indicated that the results were robust.

**Conclusion:**

The FDCS for diabetes in China was associated with a significant improvement in RE. Due to a limited time since the launch of FDCS (i.e., 2016), the recipient's physical health did not show marked improvement. In the future, FDCS should pay more attention to the physiological health of individuals with diabetes. Moreover, psychological services also need to be maintained and not slackened. At the same time, it is strongly recommended to pay more attention to the HRQoL of individuals with diabetes and more comprehensive health.

## Introduction

As a chronic and common age-related disease, diabetes has become a great challenge of worldwide public health, considering its high incidence, disability, and mortality (1). The International Diabetes Federation reported that China suffers from a great number of chronic patients, including 28% of the world's diabetics (2). Diabetes and its complications have an increasingly adverse effect on the health and quality of life of patients and increase the concern of patients regarding diet, employment, and leisure (3–5). Additionally, the nature of diabetes for patients means that these adverse effects of diabetes and its complications may be experienced for a long time (6). In this way, the quality of life of individuals with diabetes needs more attention, and more chronic disease management through primary health care is the need for those patients with long-term diabetic complications.

Primary health care is provided by family doctor contract services (FDCS), which plays a key role in the management of chronic diseases (7). In Britain, the National Health Service, founded in 1948, advocates a national management model that enables every citizen to enjoy contracted family doctor services (8). In the United States, the family doctor system originated in the 1960s and incorporated health management into community general practitioner services to provide active health management for patients with chronic diseases (9, 10). China's health care system reform starting from 2009 had given more attention and resources to primary health, which enabled it to lay a good foundation for the implementation of FDCS. In 2011, China began to take measures to establish FDCS in some pilot cities, such as Shanghai (6), and FDCS was fully launched in 2016 by the Chinese government (11). Patients with chronic diseases were listed as the focus group of the contract services considering the high incidence of chronic diseases and their adverse effects on the quality of life (12). By 2020, the goal of universal coverage of FDCS was basically achieved. With the continuous promotion and improvement of the FDCS, it was expected to have a profound influence on the quality of life of patients with chronic diseases.

Health-related quality of life (HRQoL) is the physical and mental health perceived by individuals or groups over time (13). In previous studies, it had been proved that FDCS could improve the quality and continuity of primary health care services as well as have a huge influence on the HRQoL of patients with chronic diseases (14–16). A randomized controlled trial in Norway found that the intervention of a family doctor team could effectively improve the healthy quality of life of elderly patients undergoing multi-drug therapy (17). Another study in Iran indicated that maternal and child health could be effectively improved by family doctor services (18). In addition, a study from Turkey showed that family doctor services could effectively improve the clinical outcome of diabetic patients (19). The research on family doctor services in China mainly focused on the effects for self-management, patient satisfaction, and treatment compliance (20, 21). The research on the effects of FDCS on health paid more attention to whether it could improve the health outcomes of patients. For instance, according to previous studies, FDCS could positively affect hypertension control in hypertensive patients (6). However, recently, few studies have investigated the impact of FDCS on the quality of life of diabetic patients. Therefore, this study aims to evaluate the net effect of FDCS on HRQoL in the diabetic population.

## Materials and Methods

### Data Source

The data used in this study was obtained from the follow-up survey of the China Health and Retirement Longitudinal Study (CHARLS). The CHARLS was a longitudinal survey of people over 45 years old in China, which collected high quality data representing families and individuals. The first baseline survey of the CHARLS was fielded in 2011 and the recent follow-up survey was in 2018. The 2011 baseline survey involved 17,705 practitioners, who were chosen randomly, and covered 450 villages/resident committees, 150 counties/districts, and 28 provinces. All data were collected by using face-to-face interviews and made public 1 year after the end of data collection. In general, the CHARLS is a meaningful and nice representation of the elderly in China. (A complete introduction to CHARLS is shown in Supplementary Material 1).

This study used the follow-up data from 2013 to 2018. To investigate the association between FDCS and the HRQoL of individuals with diabetes, we limited the samples to practitioners who had diabetes in 2013 and were included in the 2015 and 2018 tracking surveys. Finally, a total of 382 samples were included.

### Measure

We constructed a new scale based on the Short Form 36 (SF-36) and the CHARLS variables to measure the HRQoL of individuals with diabetes. The construction of the new scale originated from eight dimensions of SF-36, and we selected appropriate variables of CHARLS to evaluate these dimensions, including physical functioning (PF), role-physical (RP), bodily pain (BP), general health (GH), vitality (VT), social functioning (SF), role-emotional (RE), and mental health (MH) (shown in Supplementary Material 2). The construction of this scale referred to the construction methods of relevant articles which used Cronbach's α coefficient to measure the reliability between SF-36 and the CHARLS variables, and it had good reliability with all dimensions having alpha values higher than 0.6, except vitality (α = 0.34) (22). The content validity index (CVI) was evaluated at the item level (I-CVI) and scale level (S-CVI). I-CVI was higher than 0.83 and S-CVI was 0.94 (22). These indicated that the scale had good reliability and validity.

### Statistical Analysis

#### Propensity Score Matching

Propensity score matching (PSM) is applicable to non-random design and used in estimating the effect of policy interventions. The PSM method can match individuals in the control group who have similar characteristics in the treatment group with the closest propensity score. In this way, the PSM can reduce selection bias.

To evaluate the effect of FDCS on the HRQoL of individuals with diabetes in China, outcome variables between treatment and control groups were compared. But some characteristics of samples in the control group cannot consist with the treatment group. Moreover, in this study, there were more practitioners in the control group than in the treatment group. In order to solve these limitations, we used the kernel matching method, which is one of the PSM methods, to construct the control group. We also used some control covariates that were measured before the treatment, including age, gender, education, marriage, smoking, drinking, and economic situation.

The shortcoming of the kernel matching method is that the matched individuals may be bad matches. Hence, we used a visual analysis of the common support assumption of the propensity score in the treatment and control groups, which checked the overlap and the region of common support between both the groups. We then examined whether the matching procedure could balance the covariates in the treatment and control groups using a two-sample *t*-test.

#### Difference-in-Difference Model

Difference-in-difference (DID) model is widely used in policy evaluations. DID models compare changes, both in the treatment group and the control group, which attribute the effect of the policy. The FDCS can be viewed as a policy test, and DID models are adopted to analyze the net effect of this policy.

In this study, we used samples for waves 2013 and 2018 which was matched by the propensity score matching (PSM). The basic framework of DID model is given by


$$\begin{array}{l} Y_{it}=\alpha_{0}+\alpha_{1}treated_{it}^{{}^{*}}T_{it}+\alpha_{2}treated_{it}+\alpha_{3}T_{it}+\alpha_{4}X_{it}+\varepsilon_{it} \end{array}$$

where *Y*_*it*_ represents the HRQoL of individuals *i* with diabetes at time *t*; *treated*_*it*_ is a dummy variable which is equal to 1 if individual *i* received FDCS, and 0 if individual *i* did not receive FDCS; *T*_*it*_ is a time dummy variable which takes a value of 0 before implementation of FDCS, and it takes a value of 1 after implementation of FDCS; *X*_*it*_refers to other observable covariates, including gender, education, marriage, smoking, drinking, and economic situation; and ε_*it*_ is the error term.

#### Testing the Robustness of PSM-DID Estimation

The results of the robustness test are directly related to the correctness and credibility of data analysis. When we draw a conclusion, we need a series of methods to verify whether the conclusion is reliable. To ensure the robustness of the PSM-DID results, we chose the placebo test which selected samples from different periods for analysis. The FDCS was fully launched in 2016, and the 2015 data was before the implementation of FDCS. Therefore, we used the 2015 data as the treatment group for the placebo test, and we tested the robustness of DID estimation by comparing the differences between DID estimation and placebo test.

### Research Hypothesis

Based on previous studies, this study assumes that FDCS could effectively improve the HRQoL of diabetic patients.

## Results

### Characteristics of the Sample

Table 1 shows the personal characteristics of 382 practitioners. The practitioners included 58.4% (*n* = 223) women, and 91.1% (*n* = 348) were under 75 years old. A total of 72.8% (*n* = 278) of the practitioners were married and lived with their spouses, and 63.9% lived in rural areas. In addition, among the practitioners, about 41.1% (*n* = 157) had a high school degree or above, and 58.4% (*n* = 223) of whose medical insurance was the urban resident medical insurance.

**Table 1:** Characteristics of the sample (*N* = 382).

**Characteristic**	***n***	**Percentage (%)**
**Gender**		
Female	223	58.4
Male	159	41.6
**Age (years old)**		
75–85	34	8.9
55–74	251	65.7
45–54	97	25.4
**Marital status**		
Married	278	72.8
Other	104	27.2
**Residential area**		
Rural	244	63.9
Urban	138	36.1
**Education level**		
High school and above	157	41.1
Junior middle school	75	19.6
Primary school	68	17.8
Informal education	82	21.5
**Medical insurance type**		
Urban employee medical insurance	88	23.0
Urban and rural resident medical insurance	29	7.6
Urban resident medical insurance	223	58.4
New rural cooperative medical insurance	11	2.9
Other medical insurance	15	3.9
No insurance	16	4.2

### Balancing Proper Test for PSM Result

The result of balancing proper test shows that all observable covariates were balanced by the kernel matching between the treatment and control groups. The covariate difference in the two groups decreased significantly and became statistically insignificant at 5%.

**Figure 1:**
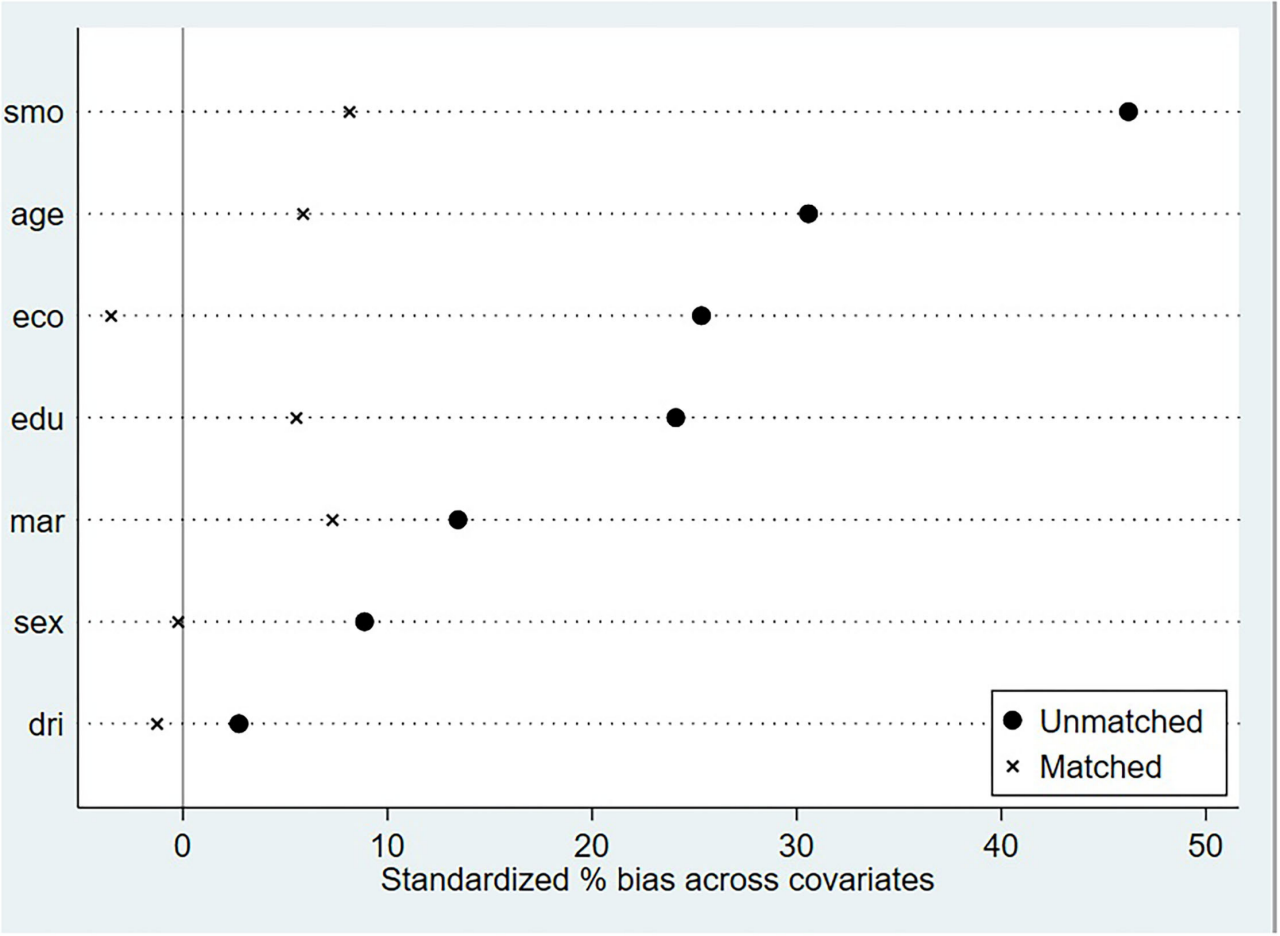
Standard deviation of covariates.

As shown in Table 2, the balancing property could be verified by comparing the significance of all matching variables in the models before and after matching. Before matching, there was a significant difference in the “smoking” and “economic situation” covariates between the treatment and control groups (*p* < 0.05). After matching, all covariates became insignificant (*p* > 0.05). The standardized bias was <10% and had reduced substantially by kernel matching (shown in Figure 1). It could also be seen intuitively in Figure 1 that the standardized bias of each covariate after matching was significantly reduced.

**Table 2:** Covariate balance test before and after matching.

**Variable**	**Unmatched/matched**	**Treated**	**Control**	***P*-value**	**Bias%**	**Reduce bias%**
Age	U	64.30	61.62	0.102	30.6	
	M	64.44	63.92	0.818	5.9	80.8
Gender (%)	U	45.45	41.49	0.623	8.9	
	M	46.88	43.45	0.992	−0.2	97.2
Education (%)	U	69.69	58.20	0.197	24.1	
	M	68.80	66.61	0.824	5.5	77.0
Marriage (%)	U	90.91	76.72	0.324	13.4	
	M	90.63	74.41	0.784	7.3	45.7
Smoking (%)	U	48.49	26.57	0.007	46.2	
	M	50.00	46.02	0.761	8.1	82.4
Drinking (%)	U	36.36	34.93	0.880	2.7	
	M	37.50	35.38	0.960	−1.3	53.2
Economic	U	91,058	27,455	0.004	25.3	
	M	33,248	42,098	0.734	−3.5	86.1

**Figure 2:**
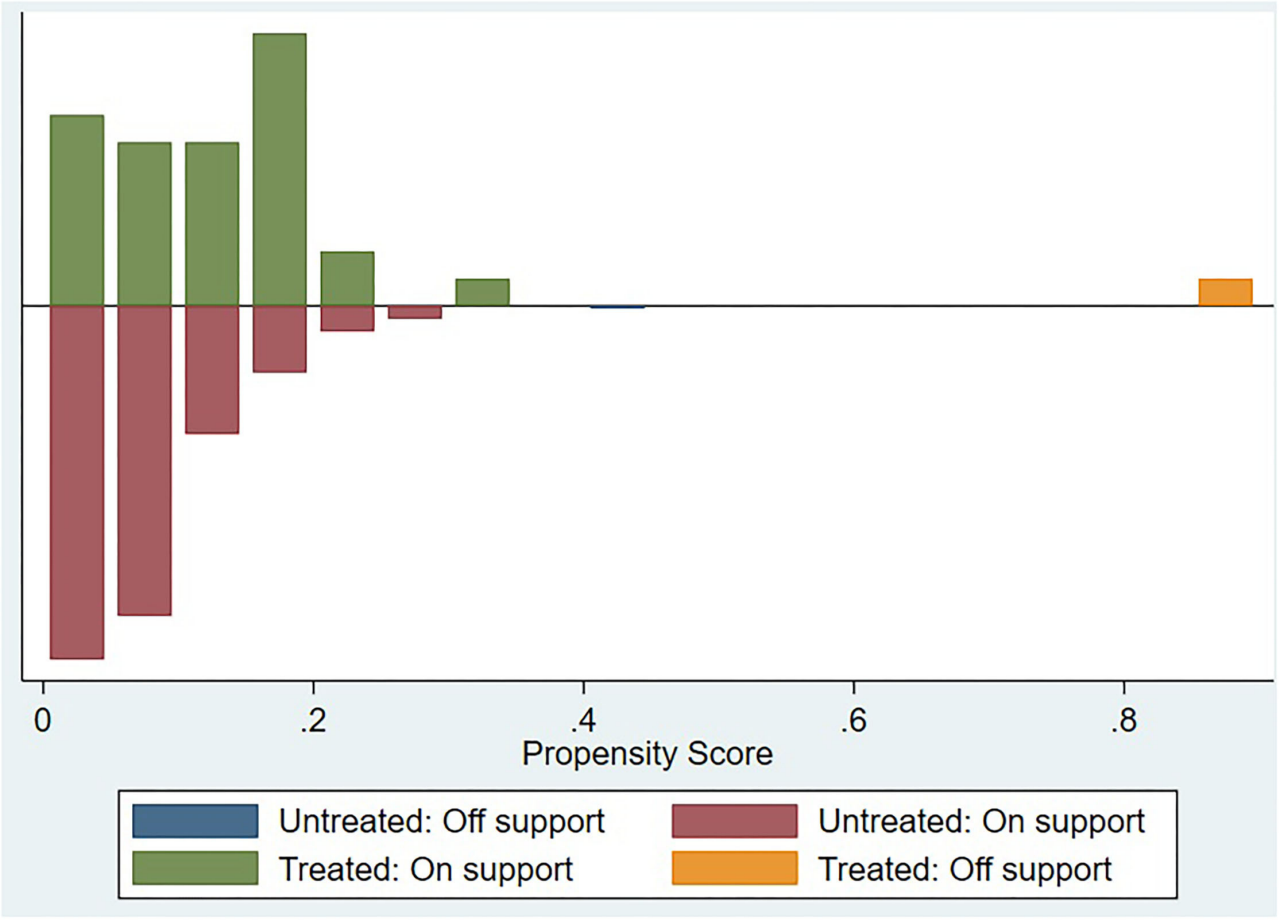
Visual analysis of the validity of the common support assumption.

Figure 2 dedicates a visual analysis of the covariate of the common support assumption in the treatment and control groups. In the figure, the treatment group is shown above the midline, while the control group is shown under the midline. The distribution of the propensity score between the treatment and control groups was similar, which confirmed the common support assumption of the covariate.

### Effects of Family Doctor Contract Services on HRQoL of Individuals With Diabetes

Table 3 shows the estimated result of PSM-DID. The “PSM-DID Estimator” in the table is the net policy effect reflecting FDCS on HRQoL of individuals with diabetes, including physical functioning (PF), role physical (RP), body pain (BP), general health (GH), vitality (VT), social functioning (SF), role-emotional (RE), and mental health (MH).

**Table 3:** Propensity score matching with difference-in-differences (PSM-DID) estimate result.

**Independent variable**	**Intervention**	**Treated mean**	**Control mean**	**Difference**	**PSM-DID estimator**
PF	Before	93.59	92.18	1.41 (0.75)	
	After	87.58	90.92	−3.34 (1.07)	−4.75 (1.32)
RP	Before	96.09	93.95	2.14 (0.65)	
	After	85.67	88.86	−0.19 (0.06)	−2.33 (0.50)
BP	Before	80.96	84.63	−3.68 (−0.99)	
	After	68.51	64.99	−3.53 (−0.02)	0.15 (0.02)
GH	Before	47.69	45.37	2.32 (0.54)	
	After	42.87	43.71	−0.85 (0.17)	−3.17 (0.50)
VT	Before	56.80	60.76	−3.96 (−0.82)	
	After	51.37	57.34	−5.97 (0.95)	−2.02 (0.25)
SF	Before	19.32	20.81	−1.50 (−0.66)	
	After	17.18	20.00	−2.82 (1.60)	−1.33 (0.44)
RE	Before	54.25	57.77	−3.52 (−0.70)	
	After	61.63	51.04	10.59^**^ (2.10)	14.10^**^ (2.02)
MH	Before	47.33	52.13	−4.80 (−1.21)	
	After	50.76	50.96	−0.19 (0.05)	4.60 (0.85)

** *P < 0.05*.

Before the implementation of FDCS, there was no significant difference in the scores of all dimensions between the treatment and control groups. After the implementation, there was a significant difference in RE. The mean score of RE increased from 54.25 to 61.63 in the treatment group, while the corresponding score decreased from 57.77 to 51.04 in the control group, and the DID value was 14.10 (*p* = 0.044). However, other dimensions had no significant difference before and after the implementation of FDCS.

### Testing the Robustness of PSM-DID Estimation

The robustness analysis of the model found that the research results were robust and reliable. The placebo test results shown in Table 4 were obtained by PSM-DID analysis of 2013 data and 2015 data. Focusing on the PSM-DID results of the RE dimension, the results showed that the difference between the treatment and control groups was insignificant. Based on the placebo test, it could be considered that the research results are robust and reliable.

**Table 4:** Robustness test results of PSM-DID model.

**Independent variable**	**Intervention**	**Treated mean**	**Control mean**	**Difference**	**PSM-DID estimator**
PF	Before	86.30	84.90	1.40 (0.72)	
	After	89.16	85.81	3.47 (1.18)	1.94 (0.55)
RP	Before	96.09	93.95	2.14 (0.65)	
	After	91.52	91.06	0.47 (0.15)	−1.68 (0.39)
BP	Before	84.47	87.60	−3.13 (−0.82)	
	After	71.26	81.22	−9.96 (1.64)	−6.83 (0.96)
GH	Before	53.70	50.51	3.19 (0.74)	
	After	80.70	70.20	10.50 (1.97)	9.31 (1.06)
VT	Before	58.06	62.44	−4.38 (−0.91)	
	After	53.42	55.42	−2.00 (0.39)	2.38 (0.36)
SF	Before	15.65	17.93	−2.28 (−1.00)	
	After	17.37	17.79	−0.42 (0.13)	1.86 (0.46)
RE	Before	75.34	76.91	−1.57 (−0.32)	
	After	74.01	77.84	−3.83 (0.26)	−2.26 (0.26)
MH	Before	51.35	55.60	−4.25 (−1.05)	
	After	55.73	55.55	0.18 (0.04)	4.44 (0.73)

## Discussion

To the best of our knowledge, most studies paid more attention to the influence of FDCS on physiological indexes of individuals with diabetes, while few studies paid attention to its effect on HRQoL, especially the specific dimension of HRQoL. In this way, this study assessed changes in each dimension of HRQoL of individuals with diabetes to evaluate the net effect of FDCS. The results demonstrated that FDCS effectively improved the scores of RE in individuals with diabetes, but not that of MH, VT, SF, PF, RP, BP, and GH.

According to the results, FDCS for individuals with diabetes significantly improved the scale scores of RE. This result is similar to the finding by Moffatt (23), who reported that care for chronic diseases effectively raised the population's RE score. Moreover, FDCS could provide high-quality home care services for patients with chronic diseases and bring convenience to patients with inconvenient actions. According to a previous study, RE reflects role impairment due to emotional distress in aspects of work or task performance as well as the degree of satisfaction with achievements (24). Since the sample population in this study was mostly the elderly who had retired, RE more reflects the emotional distress and satisfaction when completing certain tasks or behaviors in life. According to Sabbah's research (25), FDCS could reduce the occupancy rate of nursing homes and hospitals and maintain the independent living ability of the elderly. Therefore, the elderly who signed up to receive FDCS had higher satisfaction when completing certain tasks or behaviors in their life, and the RE value was higher.

Although FDCS significantly improved the RE of individuals with diabetes, it did not improve the MH, VT, and SF. A similar situation was also revealed in a previous study, which showed that psychological well-being was more related to the quality of life, compared to physical health and socioeconomic status (26). According to the WHO report, mental health is the fifth leading cause of disability-adjusted life years among the elderly in China (27). However, FDCS did not pay enough attention to mental health, and there was no routine mental health examination for individuals with diabetes (28). Additionally, despite being both mental and physical health very important for the quality of life of individuals with diabetes, FDCS providing primary health care services usually paid more attention to the physical health of residents than mental health (29). In terms of the mental health, although role-emotional has improved significantly, the other three dimensions are still not improved. Therefore, FDCS need continue to pay attention to the other three dimensions of diabetic patients.

This study showed that the contract service did not improve PF, RP, BP, and GH. For individuals with diabetes, the progression of diabetes is a slow process. In this way, the result may be explained by the finding of Behma's study (30), which indicated that a postponed progression of morbidity and symptom may not directly affect the self-rated health of elderly patients, and there are differences between elderly patients' perception of health and satisfaction with health. Thus, due to this cognitive bias toward health, patients with chronic diseases may also have certain errors in their own health evaluation. Moreover, since FDCS was implemented after 2016, the progression and physical health of individuals with diabetes could also not be significantly improved in the short period of 2 years. Additionally, another explanation could be connected to a stereotypic view that “be old is to be ill”. This view shows that although the physical condition is deteriorating, the elderly patients are satisfied with their overall health status (31). Although FDCS has paid much attention to the physiological health of individuals with diabetes, the four dimensions of physiological health of the population have not been significantly improved. In the future, FDCS should pay more attention to the physical health of individuals with diabetes. At the same time, service on physical health needs further adjustment and optimization. Above all, FDCS had no significant effect on the health of diabetic patients at present, but the effect could be expected over a longer period.

In comparison with previous studies, this study is strengthened by two features (14–19). First, the data for this study were obtained from CHARLS, which is a national database with good representation. Second, this study uses PSM-DID to evaluate the causal relationship between FDCS and HRQoL, which could effectively eliminate the influence of external confounding factors on evaluation. Moreover, some limitations of this study should be recognized. On the one hand, since the latest CHARLS data of 2021 has not yet been published, this study selected the CHARLS data of 2013 and 2018 to evaluate the net effect of FDCS on HRQoL of individuals with diabetes. On the other hand, only those individuals with diabetes receiving all follow-up surveys in 2013, 2015, and 2018 were included in this study, which made the sample size of this study relatively small, but these samples are from the whole national data of CHARLS without additional elimination.

## Conclusion

In this study, we evaluated the net effect of FDCS on eight dimensions of the HRQoL of individuals with diabetes. The findings showed that the FDCS probably has an influence on the HRQoL by significantly improving the RE. However, the improvement of the other seven dimensions of HRQoL was not significant. In the future, more attention should be paid to FDCS to the physiological health of patients with diabetes, and the frequency of regular follow-up visits by family doctors should be increased. Additionally, since the other three dimensions of mental health did not improve significantly, psychological services need to be maintained and not slackened. It is also recommended to pay more attention to the HRQoL of individuals with diabetes and more comprehensive health.

## Data Availability Statement

The datasets presented in this study can be found in online repositories. The names of the repository/repositories and accession number(s) can be found below: http://charls.pku.edu.cn/.

## Ethics Statement

The studies involving human participants were reviewed and approved by the Research Ethics Committees of Peking University (IRB000010529611015). The patients/participants provided their written informed consent to participate in this study.

## Author Contributions

WL and LW contributed to the conception and design of the study. LW conducted the data reduction and analyses and also wrote the manuscript. WL guided the whole process and reviewed the manuscript. All authors read and approved the manuscript before submission.

## Funding

This research was supported by the Distinguished Young Scientific Research Talents Plan in Universities of Fujian Province (Grant No: 2018B030). The funders had no involvement in study design, data collection, statistical analysis, and manuscript writing.

## Conflict of Interest

The authors declare that the research was conducted in the absence of any commercial or financial relationships that could be construed as a potential conflict of interest.

## Publisher's Note

All claims expressed in this article are solely those of the authors and do not necessarily represent those of their affiliated organizations, or those of the publisher, the editors and the reviewers. Any product that may be evaluated in this article, or claim that may be made by its manufacturer, is not guaranteed or endorsed by the publisher.
