# Supplementary material for: Effects of Family Doctor Contract Services on the Health-Related Quality of Life Among Individuals With Diabetes in China: Evidence From the CHARLS
Source: Front Public Health. 2022 May 4;10:865653. doi: 10.3389/fpubh.2022.865653 (PMC9114482; doi:10.3389/fpubh.2022.865653)
Supplement: Supplementary file 1 [file Table_1.DOCX]

**Introduction of CHARLS**

The China Health and Retirement Longitudinal Study (CHARLS) aims to collect a high quality nationally representative sample of Chinese residents ages 45 and older to serve the needs of scientific research on the elderly. The baseline national wave of CHARLS is being fielded in 2011and includes about 10,000 households and 17,500 individuals in 150 counties/districts and 450 villages/resident committees. The individuals will be followed up every two years. All data will be made public one year after the end of data collection. CHARLS adopts multi-stage stratified PPS sampling. As an innovation of CHARLS, a software package (CHARLS-Gis) is being created to make village sampling frames.

CHARLS is based on the Health and Retirement Study (HRS) and related aging surveys such as the English Longitudinal Study of Aging (ELSA) and the Survey of Health, Aging and Retirement in Europe (SHARE). The pilot survey of CHARLS was conducted in two provinces (Gansu and Zhejiang) in 2008 and collected data from 48 communities/villages in 16 counties/districts, covering 2,685 individuals living in 1,570 households. The response rate of the pilot survey was 85%.

The CHARLS questionnaire includes the following modules: demographics, family structure/transfer, health status and functioning, biomarkers, health care and insurance, work, retirement and pension, income and consumption, assets (individual and household), and community level information.

CHARLS has received critical support from Peking University, the National Natural Science Foundation of China, the Behavioral and Social Research Division of the National Institute on Aging and the World Bank.

If you’re interested, CHARLS can be found here: http://charls.pku.edu.cn/
