# Supplementary material for: Effects of Family Doctor Contract Services on the Health-Related Quality of Life Among Individuals With Diabetes in China: Evidence From the CHARLS
Source: Front Public Health. 2022 May 4;10:865653. doi: 10.3389/fpubh.2022.865653 (PMC9114482; doi:10.3389/fpubh.2022.865653)
Supplement: Supplementary file 2 [file Table_2.docx]

Corresponding variables in the CHARLS data

| HRQoL |  | CHARLS validity |
| --- | --- | --- |
| **PF** |  | db001 db002 db003 db004 db005 db006 db007 db008 db009 |
| **RP** |  | db016 db017 db018 db019 db020 |
| **BP** |  | da041 da042s1 da042s2 da042s3 da042s4 da042s5 da042s6 da042s7 da042s8 da042s9 da042s10 da042s11 da042s12 da042s13 da042s14 da042s15 |
| **GH** |  | da001 da002 |
| **VT** |  | dc015 dc018 |
| **SF** |  | da056s1 da056s2 da056s3 da056s4 da056s5 da056s6 da056s7 da056s8 da056s9 da056s10 da056s11 da056s12 |
| **RE** |  | dc010 dc012 |
| **MH** |  | dc009 dc011 dc014 dc016 dc017 |
